# A First Attempt to Bring Computational Biology into Advanced High School Biology Classrooms

**DOI:** 10.1371/journal.pcbi.1002244

**Published:** 2011-10-27

**Authors:** Suzanne Renick Gallagher, William Coon, Kristin Donley, Abby Scott, Debra S. Goldberg

**Affiliations:** 1 Department of Computer Science, University of Colorado, Boulder, Colorado, United States of America; 2 Boulder Valley School District, Boulder, Colorado, United States of America; University of California San Diego, United States of America

## Abstract

Computer science has become ubiquitous in many areas of biological research, yet most high school and even college students are unaware of this. As a result, many college biology majors graduate without adequate computational skills for contemporary fields of biology. The absence of a computational element in secondary school biology classrooms is of growing concern to the computational biology community and biology teachers who would like to acquaint their students with updated approaches in the discipline. We present a first attempt to correct this absence by introducing a computational biology element to teach genetic evolution into advanced biology classes in two local high schools. Our primary goal was to show students how computation is used in biology and why a basic understanding of computation is necessary for research in many fields of biology. This curriculum is intended to be taught by a computational biologist who has worked with a high school advanced biology teacher to adapt the unit for his/her classroom, but a motivated high school teacher comfortable with mathematics and computing may be able to teach this alone. In this paper, we present our curriculum, which takes into consideration the constraints of the required curriculum, and discuss our experiences teaching it. We describe the successes and challenges we encountered while bringing this unit to high school students, discuss how we addressed these challenges, and make suggestions for future versions of this curriculum.We believe that our curriculum can be a valuable seed for further development of computational activities aimed at high school biology students. Further, our experiences may be of value to others teaching computational biology at this level. Our curriculum can be obtained at http://ecsite.cs.colorado.edu/?page_id=149#biology or by contacting the authors.

## Author Summary

We have designed and implemented a curriculum to teach basic computational biology to advanced high school students. The curriculum includes an introduction to the concept of algorithms, an overview of the Basic Local Alignment Search Tool (BLAST) algorithm used to compare DNA sequences, and methods for building phylogenetic trees. We taught this curriculum in advanced biology classes at two local high schools. As a result of this, we were able to give many students an appreciation of the role computers play in biology and an idea of why computational methods are needed in biological research. We found that while the high school students lacked the necessary background in math and computer science to be able to write their own algorithms, they were able to use existing algorithms, analyze them, and compare the results. We also encountered a number of challenges that could arise in other attempts to teach computational biology to students at this level, whether using our curriculum or another. We discuss each of these challenges and possible ways that they can be overcome.

## Introduction

The absence of a computational element in secondary biology classrooms is of growing concern to the computational biology community. While computer science is increasingly important in modern biology research [1]–[4], it plays almost no role in high school and undergraduate biology classes. Few of these students are even aware that computation plays any role in biology [5]. We developed a computational biology unit aimed at advanced high school students and taught this unit in three different classes in two local high schools. The goal of this unit is to teach students the connection between computer science and biology and demonstrate how computational techniques can lead to biological discoveries.

This project was undertaken as part of the Engaging Computer Science in Traditional Education (eCSite) Program, which is funded by the National Science Foundation (NSF) GK-12 program. The goals of the GK-12 program include training graduate students to communicate their research to the general public while providing K-12 students a glimpse of the work and life of scientists. eCSite is a 5-year program that began in 2009 to bring computer science into traditional K-12 classrooms. The goals of eCSite include improving computational literacy, which is important to all people living in the 21st century, and raising awareness of the possibilities for impacting our world with a career in computer science by showing K-12 students and teachers some of the ways computing affects modern life. Rather than create new computer science classes that only students already interested in computer science are likely to take, the goal is to bring computer science into classes such as geography, physics, government, art, and biology.

In our first year, two eCSite fellows (graduate students in computer science) worked with Advanced Placement (AP) Biology teachers at two different high schools (as described below, the content was also brought to other advanced high school students taught by these teachers). The eCSite fellows and teachers developed and presented a unit to help students gain a deeper understanding of genetic evolution (a requirement in the AP biology curriculum) using the Basic Local Alignment Search Tool (BLAST) [6] and phylogenetic trees in three distinct lessons. In the first lesson, we gave a brief introduction to algorithms. In the second, we gave an introduction to BLAST, including demonstrating the sequence alignment problem and the challenges associated with it. In the third lesson, we showed them an application of BLAST, building evolutionary trees using the matched sequence.

Our lessons were largely successful in showing students the relative scale of computation involved in genomic research and giving them an appreciation for the methodology that goes into any computational research. Students were able to complete the lessons and gain an understanding of how algorithms such as BLAST might be used in biological research and why some understanding of these algorithms is important to students of biology. While few students had the necessary background to construct complex algorithms, they were able to use and analyze existing algorithms.

Teachers reported that student reaction to the curriculum was mixed. Some students were interested in the computational element, seeing it as a tool used by “real” biologists and eager to learn more about how it was used. These students, upon seeing the amount of genetic data available in the various databases, realized that advances in this area would be impossible without good computational methods to analyze the data. Other students, however, either failed to see the importance of computation to their current studies or indicated that they did not have time to learn about something that would not explicitly be on the AP or International Baccalaureate (IB) test. We discuss these challenges as well as possible ways of overcoming them below.

Despite these challenges, we believe that our curriculum can serve as a starting point for further development of computational biology activities for high school students. We also believe that our experiences, including our difficulties and our methods of overcoming them, will be of value to other computational biologists interested in updating the content of high school biology classes.

## Methods

### Curriculum Development

Two eCSite fellows (computer science graduate students) with research experience in computational biology worked closely with two AP biology teachers in order to introduce computational methods into required AP biology lessons. Beginning in September, they met weekly to brainstorm possible units in an effort to minimize time taken away from required content. In addition, the two eCSite fellows met with each other on a weekly basis to coordinate the planning with both teachers, and monthly with an eCSite co-director whose research interests are in computational biology to help guide the content. There were periodic meetings of all involved (the eCSite fellows, AP Biology teachers, and co-director). Curriculum was developed during the fall semester and delivered to the classes during the spring semester.

### The Curriculum

Our curriculum consisted of three basic lessons: an introduction to algorithms, a basic discussion of the BLAST algorithm [6], and algorithms to construct phylogenetic trees.

The first lesson, an introduction to algorithms, was designed to introduce students to the basic vocabulary involved with algorithms and get students thinking algorithmically. We started by introducing students to algorithms for every day tasks, such as making coffee, then asking them to write their own algorithms to make peanut butter sandwiches. The next activity had students participating in a “living computer” algorithm: each student was given a numbered instruction that represented one step in the algorithm. Students then stood in line in front of the class in the order that the steps would be performed in the computer. Each student performed their step and handed the “output” to the next student in line until the final step had been reached. After each step was acted out, it was executed on a computer using the same inputs so that the students could see the same output coming from the computer as their “living computer”. The students executed the algorithm, and then were shown the computer code that did the same thing. This allowed students to see what sort of basic steps should be included in an algorithm and how those steps could be translated to computer code. Finally, we had students write algorithms to create Punnett squares, diagrams to predict the outcome of a cross-breed given the genotypes of the parents. Students were already familiar with the creation of Punnett squares, so this helped students think about biological problems in algorithmic terms.

**Figure 1:**
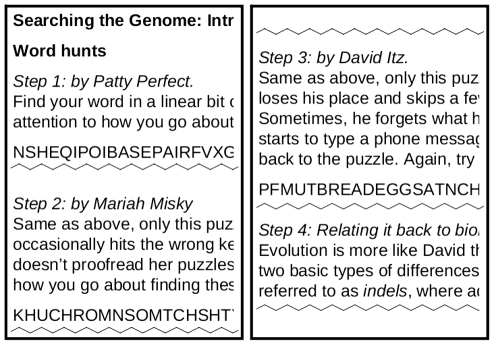
Introducing students to BLAST. Students are introduced to the problems inherent in searching the genome with a series of word search puzzles designed to model a genome with no mutations, a genome with SNPs, and a genome with both SNPs and indels.

The second lesson focused on DNA sequence comparison using BLAST. This lesson had two parts. In the first part of the lesson, we explained BLAST in terms of a word search. We gave students three word search puzzles containing various vocabulary words from biology and genomics (see Figure 1). The first was a “perfect” word search, simulating the problem of finding an unmutated gene in another genome. In the second puzzle, students were given a “wrong key” word search created by someone who would occasionally hit the wrong key, so some letters might be wrong. This served as a simulation of the problem in a genome with single nucleotide polymorphism (SNP) mutations. The third puzzle was designed to simulate the problem in a genome that contains both SNP and insertion/deletion (indel) mutations. Students were given a “ditz” word search that contained not only wrong letters, but missing or added letters, as if the person writing the word search may have skipped parts of the puzzle or forgotten what he was doing and started typing in a shopping list or telephone message. The second part of the lesson introduced students to an implementation of the BLAST algorithm. We asked students to choose a disease with a genetic basis, then search for that gene in the National Center for Biotechnology Information (NCBI) database. Once they obtained the DNA sequence for that gene, they were asked to run it through the BLAST implementation on the NCBI website to find similar genes in humans and other species. Students were then asked to answer several questions about the BLAST results, including: give the scientific and common names of species that have similar genes, give the percent of base pairs that could be matched in a given alignment, and give an example of an alignment that contains an indel.

The third lesson was building phylogenetic trees. Students were given sequence data on the *COX15* gene from eight different species. These genes were identified using only first names (“Alex”, “Chris”, etc.) without any information about what species they came from. Students were then asked to construct a phylogenetic tree by running pairwise BLAST to compute a similarity score between every pair of genes, and to cluster based on these results. Students were taught a simple hierarchical agglomerative clustering algorithm [7], and different students were given different metrics for determining distance between clusters: some students were asked to cluster by considering the distance between clusters to be the distance between the closest two points in the clusters (single linkage), some students were asked to consider the distance between clusters to be the distance between the furthest two points in the clusters (complete linkage), and the remainder were asked to consider the distance to be the average distance between all points in the clusters (average linkage). Students then compared the results of their different clustering algorithms to see that seemingly minor differences in algorithm implementation can result in different biological conclusions, reinforcing the notion that biologists need to understand details of the computational tools they use, even if they aren't themselves developing the tools. Finally, students used BLAST to identify species from their sample genomes. Once species were identified, students could compare their results to their predictions of species relatedness.

### Teaching

This unit was taught in three different classes in two different high schools: Centaurus High School and Monarch High School. Centaurus is a high school located in Lafayette, Colorado, with 28% of students receiving free or reduced lunches. Monarch is a high school in Louisville, Colorado, with 4% of students receiving free or reduced lunches (data is from the Boulder Valley School District website at http://www.bvsd.org/and is based on numbers collected in October 2009). In Centaurus High School, the unit was taught to a combined AP/IB Standard Level (SL) Biology class composed mostly of high school juniors with some seniors. At Monarch High School, it was taught to an AP Biology class and a dedicated Biotechnology science elective class. There was little overlap between students in the Biotechnology class and those taking any AP science classes. Due to scheduling constraints in the schools, the lessons in the unit were spread out over a period of approximately three months. Lessons were taught primarily by the eCSite fellows in collaboration with the classroom teachers. For various reasons, not all activities were done in all classrooms.

Lessons were evaluated through discussion with the classroom teachers. In formal and informal discussions, the teachers reported to the fellows their observations about which part of the lessons the students had enjoyed, which parts they had struggled with, and whether or not students were able to connect these lessons to other lessons from biology class. Teachers also reported on the general types of comments, questions, and complaints they had received from students about the curriculum.

## Results/Discussion

Teachers reported a variety of reactions from the students. Many students were enthusiastic about learning about BLAST, a tool used by “real” scientists. These students saw this lesson as providing a connection not only between biology and computer science, but between what they were doing in class and the research done by working biologists. Other students, however, saw this unit as at best irrelevant and at worst a distraction from more important lessons.

In general, students were unable to complete assignments that involved algorithm creation, even ones as simple as “Write down detailed instructions about how you solved this problem.” The students were seemingly able to understand and reproduce the algorithms for “every day” tasks such as getting coffee or making a sandwich, but they were unable to transfer these skills to more complicated problems such as solving the Punnett squares or word searches.

Students were more successful, however, at understanding existing algorithms. The “living computer” activity was successful, with students interested in how their instructions combined in order to complete a calculation, even though none of them knew what the goal of the calculation was ahead of time. They were also interested to see how the human instructions they were given translated to computer code. Similarly, there was strong interest in how the different algorithms used to build the phylogenetic trees created different trees.

Not all students found the layout of the NCBI website to be intuitive. Some groups had more difficulty navigating the NCBI website than we expected. In particular, in the second lesson some students had difficulties searching for genetic diseases, then finding the sequences for the associated genes. Students also seemed uncomfortable with the “open-ended” nature of that activity, and many said that they would have preferred a more structured activity with a specific disease and gene to study.

### Successes

Our activities were generally successful and had the students interested and engaged.

Most students enjoyed the first lesson. They liked seeing an algorithm on how to get a cup of coffee and making their own algorithms to make peanut butter sandwiches. They also enjoyed the living computer activity and seeing how an algorithm worked and translated into computer code. This suggests that there is interest in computational and algorithmic activities as long as these activities are presented at the correct level.

**Figure 2:**
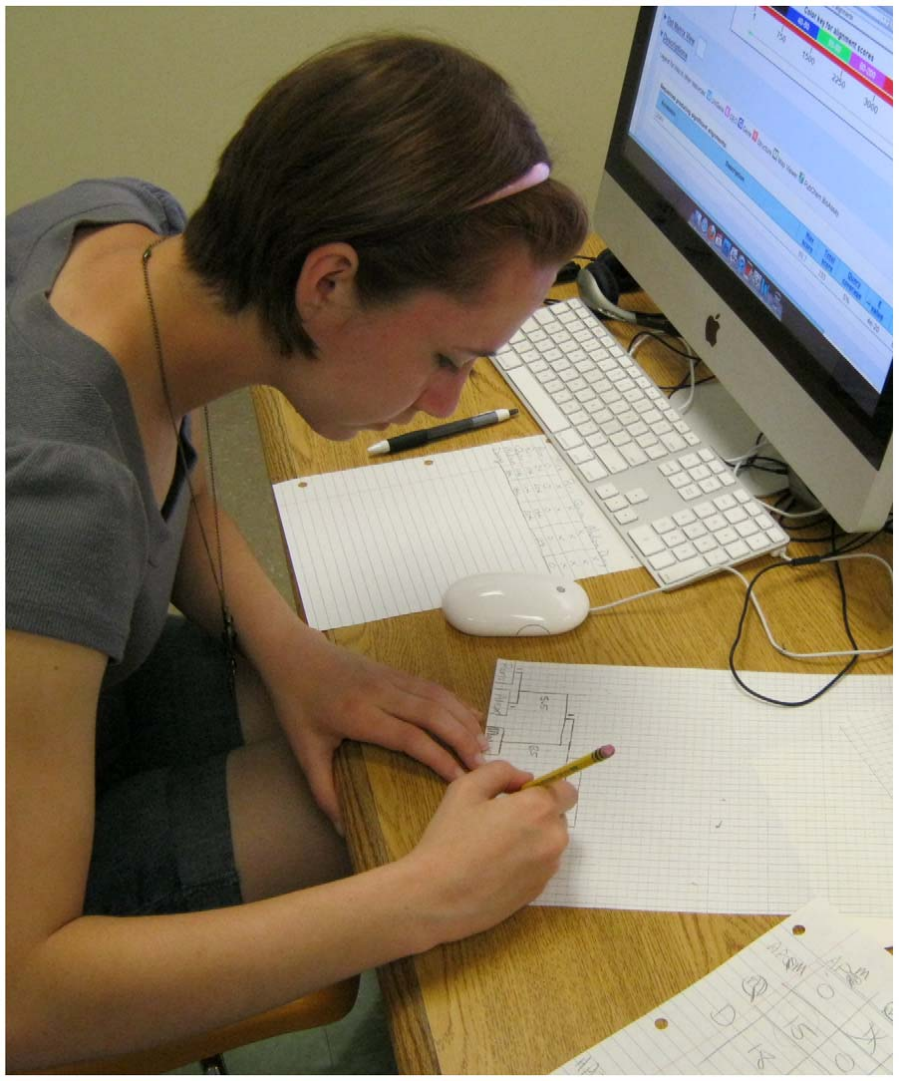
A student builds a phylogenetic tree as part of the third lesson.

In the later, more biology-oriented lessons, students enjoyed doing the word search puzzles. Likewise, they seemed interested in seeing how different hierarchical clustering algorithms created different phylogenetic trees (Figure 2). Also in the third lesson, they seemed to enjoy trying to figure out from which species their genome sequences came. Again, we found that as long as students were readily successful at the task at hand, they were willing to engage with the material.

The unit was a success in that most students were able to complete the assigned activities. Though there were multiple difficulties and challenges (detailed below), the vast majority of students were able to run a genetic sequence through BLAST and find multiple species with similar genes. They were able to use BLAST to compare two genetic sequences and build a phylogenetic tree using the results.

#### Student learning about BLAST

Students learned about BLAST and what types of information it could and could not convey. Students compared BLAST to a previous technique they had studied in class, restriction fragment length polymorphism (RFLP) analysis in forensic investigations, and discussed the difference between the two methods of comparing different sequences.

Students already had an understanding of genetics, and once they got some experience with the vast amount of genomic data that needs to be manipulated and analyzed, it became clear to them that gaining any meaningful knowledge in this field would be impossible without employing some form of computational methodology. Once they applied the algorithms in a real-world application, they also understood how computer science is used by biologists throughout the world.

#### Student learning about phylogenetic trees

Students were able to look at pairwise BLAST results and recognize which ones indicated closer relationships between species. They learned three different algorithms for hierarchical clustering and were able to compare the final trees created by each algorithm. As a result of looking at the different trees, they gained some understanding of why it is important for those interested in biology to know something about the algorithms used to create them. Students also gained a deeper understanding of evolutionary relationships and how they can be determined, a concept tested in AP exams.

#### Teacher learning about computational biology

In addition to helping students learn about computational biology, teachers were introduced to computational resources used in modern biology research and how these might be used to enhance a traditional biology curriculum. While both teachers were reasonably tech-savvy and enthusiastic about using computers in the classroom (hence the reason that they joined the program), fellows were still able to show them new computational resources. While the teachers had some familiarity with BLAST, they appreciated seeing an implementation of the algorithm and having an explanation of the outputs. They also appreciated the assistance in developing a formal lesson plan to introduce BLAST to their students and use it in the classroom.

Teachers also learned how computation could be used in lessons that were already part of the curriculum. Lessons on genetics, evolution, and phylogenies were already being taught. Together, the teachers and fellows determined how BLAST could enhance understanding of genetics, show evidence for evolution, and give students an idea of the methods behind the creation of the phylogenetic trees they were studying.

### Challenges

Over the course of the unit we encountered several challenges. We detail these here along with possible responses to them.

#### “I'm interested in biology, not computer science. Why should I care about this stuff?”

We expected that many high school biology students would not see the connection between biology and computer science prior to the start of the unit. We also were not surprised to learn that few students saw the connection between the first lesson, the general introduction to algorithms, and biology. Many students found the connection once we started working with BLAST; once these students saw how the algorithms could be used to manipulate the vast amounts of genetic data, they quickly saw the importance of computer science to biology. There were some students, however, who persisted in thinking of the lesson as just “computer stuff,” irrelevant to the rest of biology, even after the BLAST lessons began.

Part of the difficulty here, we believe, is the fact that the lessons were taught by the fellows, whom the students knew were computer scientists rather than biologists. We believe that this problem may be aided by bringing working biologists who deal with computational aspects into the classroom. In addition, we would like to work more closely with the classroom teacher and try to tie our first lesson on algorithms more closely to other activities that are already part of the biology curriculum; for example, by having the “living computer” algorithm be for a Punnett square computation, which is part of the AP curriculum.

#### “But as long as these algorithms work, why do I need to understand how they work? I can just use them.”

Even among students who acknowledge the connection between biology and computer science, there was resistance to learning about BLAST and other computational biology algorithms. The reasoning went like this: “These programs might be useful, but other people have already written them. I might use them, but I'm not planning to try to improve them or write any others. So why learn about the computation when I can just use the existing programs?”

The solution to this challenge was to show students places where a greater understanding of the underlying algorithm leads to a better use of existing programs. The phylogenetic tree lesson was designed with confronting this challenge in mind. From a computer science perspective, all three trees produced were equally correct. However, they are not identical, and from a biology perspective one may be preferable to others. A biologist's perspective allows for a correct clustering algorithm to be selected. In addition, a biologist using the algorithm who understands the different options in clustering techniques is more likely to recognize a potential trouble spot and be able to guide the choice of algorithm parameters to lead to better results.

We believe that similar techniques could be incorporated into future versions of earlier lessons. For example, the BLAST lesson could involve further discussion of the parameters, such as scoring matrix and seed length, and how they affect results.

#### “This isn't going to be on the AP test, and I need to study for that, so I can't learn it now.”

There is a great deal of controversy about the merits of high-stakes testing. Whether these tests are a good or bad idea, however, is irrelevant for our purposes; they are a reality, and a reality that those teaching in the high schools must deal with.

We were aware of this while developing our unit and worked to incorporate our lessons into the existing curriculum. What we had underestimated, however, was the response of the students themselves. Teachers reported that several students were concerned about the upcoming AP and IB tests, the financial investment they and their parents had made in paying for the tests, and the benefits that they hoped to reap from a good performance on these tests. It was evident that the exams were a major motivating factor for them, and that they resented what they saw as a “distraction” from test preparation.

Long term, the best solution to this issue would be to incorporate a computational biology/bioinformatics element into the AP and IB tests. In the short term, however, it is still possible to incorporate computational elements into an AP class without having them seen as a distraction from the test by teaching things already on the test using computational methods. For example, students learn how to make Punnett squares, and we believe thinking about this task algorithmically helps students understand this more deeply. Also, one of the AP Biology requirements is to be aware of some of the evidence for evolution; one piece of evidence is the similarity of genetic sequences across species, something that can be taught using BLAST. The test asks for knowledge of the genetic basis of some diseases, something that might be taught by BLASTing disease-causing genes. The exam has questions on metabolism, some of which might be answered using basic metabolic networks. By teaching things on the test using computational methods, and possibly showing students questions from past exams that relate to the work they have done, we can show them that understanding the computational element can be an asset rather than a distraction in their exam goals.

It should be noted that this challenge primarily arose in the first high school in which this lesson was taught, with few if any of the students at the other school voicing concerns about the distraction from the tests. One hypothesis that might explain this difference is that there were more technical difficulties in the lesson at the first school, causing students to have more fears about “wasting time” while the difficulties were ironed out. However, we wished to mention this issue as something that might arise in teaching a computational biology lesson in an AP or IB class.

### Using the Curriculum

The curriculum is designed to be taught by computational biologists interested in updating the content of high school biology classes. While the fellows who taught this particular curriculum were embedded in the classroom for most of the school year, this would not be necessary. The lessons are designed to be done in one or two class periods and could be taught either by a computational biologist coming into the classroom for a few days or as part of the high school outreach science camps that many colleges offer during weekends or the summer.

While the lessons are designed to be introduced by a computational biologist, we hope that after team-teaching the lessons with a high school biology teacher, the teacher will be able to teach the lessons without assistance. The lessons do not require the teacher to know how to program; the computer portions of the lessons use either pre-existing, publicly available tools (such as the implementation of BLAST on the NCBI website), or programs that are already written and can be downloaded along with the curriculum. One of the two biology teachers involved in the first year of our program successfully taught the lesson independently in the second year. In addition, since high school biology teachers helped develop these lessons so that they integrate well with the required AP Biology curriculum and address required “essential knowledge,” teachers who are motivated to incorporate modern methodology and are comfortable with computers may be able to teach them independently from the beginning.

The curriculum can be used as is; however, we encourage those using the curriculum to modify it, either to suit their own needs, to correct potential flaws in the curriculum (see below), or otherwise improve it. The curriculum that we have developed is a first attempt to bring a computational element into the high school biology curriculum, not a final product. It is a functional lesson plan and could be used by those not interested in developing their own curriculum, but there is much room for improvement by those interested.

### Assessment of the Curriculum

Based on our experience and feedback from other computational biologists, we believe there are several areas where a review of the current lessons would be of value. Some lessons would benefit from changes in the activities. Other lessons need a more thorough explanation of how the lesson ties into the broader picture of biological research. Though simplifications are appropriate for the high school lesson, we would highlight these simplifications to show students how researchers would do things differently.

In reviewing the first lesson, the general introduction to algorithms, our first question was, “Is this lesson necessary? Do the activities involving general algorithms enhance the understanding of the later, biological algorithms, or are they an unnecessary sideshow?” Critics of the first lesson felt that it should be eliminated because it is largely unrelated to biology and makes students more inclined to see the entire unit as a distraction. Countering that view is the argument that BLAST and phylogeny algorithms represent breakthroughs in computer science as well as biology, and students need to understand something about algorithms in order to get the “big picture.” Most people involved in the development of the curriculum, including one of the two high school biology teachers, felt that the algorithms lesson was important, though it could use some improvements to tie it more closely to the study of biology.

We plan to eliminate the activity where students were asked to develop the algorithm for Punnett squares. Our experience indicated that this activity was too difficult for most of the students and generally produced frustration rather than understanding. In order to accomplish the goal of the activity, to get students to think about biological problems in algorithmic terms, we may instead show students an algorithm for Punnett squares using the “living computer” or some other activity.

We also discussed changing the order of the lessons in order to move the general discussion on algorithms from the first lesson to a place where students might better appreciate the connection between algorithms and biological research. One possible placement would be after the word search puzzles given in the lesson on searching for similar DNA sequences. After students complete the first (or all three) word search puzzles, students could be asked to describe general strategies that could be used for word search. Strategies could include looking for each word separately, looking for the start of all words simultaneously in the first pass of the word search, and looking through the word search for sequences that seem “word-like” without first reading the list of hidden words and then scrutinizing locally around these areas more carefully. Our experience indicates that such an approach would need to be scaffolded with ideas from the high school teacher, because even after a complete lesson on algorithms, students had difficulty describing their word search strategies when asked. Students could be asked what they read first (the word search or the list of hidden words). After coming up with different general approaches, the lesson would progress to details of an algorithm, perhaps in living algorithm format. Alternatively, the discussion on algorithms could be placed after both the lesson on searching DNA sequences and building phylogenetic trees. This would allow students to learn about algorithms after they have already seen the algorithms for BLAST and building phylogenetic trees. Examples would then be drawn from both these problems rather than from general tasks, making the lesson seem more relevant.

Our review of the second lesson, the lesson on BLAST, showed two places where we feel changes would be beneficial. The first was that the “open-ended” nature of the computer activity had students feeling confused and directionless, especially those students who had trouble navigating the NCBI website. We have modified the lesson so that rather than looking for any genetic disease, all students look at sickle cell anemia. We have also included the code for the hemoglobin beta gene in both rat and human so that students would not need to navigate the website in order to find the correct code. These changes are already reflected in our curriculum posted online.

In order to reinforce the idea that biologists whose work does not require programming still need to understand how computational tools work, we would introduce an element into the second lesson to use different BLAST parameters. We would have students try different seed lengths, different match/mismatch scores, and different gap costs. We would like students to come away from the lesson with a sense not only of the problem BLAST is trying to solve and how the program can be useful, but also how knowing more about the BLAST algorithm can aid them as biologists. As we add to this lesson, however, we must be mindful of the additional time it would take and whether or not this time can be justified given the rigid constraints of the AP and IB course requirements.

Our review of the third lesson, building phylogenetic trees, focused primarily on whether or not we were misleading students about the methods used by biologists. We ask students to build evolutionary trees using a clustering procedure based on sequence similarity. While this, in broad strokes, is what evolutionary biologists would do, the methods are not up to standard. BLAST does not give a good distance metric for phylogenetic trees, and our clustering methods are all based on the unweighted pair-group method using arithmetic averages (UPGMA), which is known to perform poorly in some cases. However, our initial methods have two relevant advantages. Using BLAST rather than another method of sequence similarity lets us build on the second lesson and gives students more practice using BLAST. Also, by using a tool with which the students were already familiar, we avoided having to teach another tool. While some tools for building phylogenetic trees from sequence data are relatively easy to learn, if we were to use a different tool for determining similarity, we would have to either teach students a general understanding of the underlying algorithms as well as how to use that tool, or give them a pre-computed matrix of similarity scores. The former option might take time that is not available within the course constraints, while the latter option would deny students the opportunity for further practice with a computational element. Introducing the concepts of parsimony, maximum likelihood, and Bayesian inference would likely take too much additional class time, given that these concepts are not tested on the AP or IB exams. On the other hand, the simple UPGMA clustering algorithms require little teaching time and are easy for the students to both do and understand, while more complicated methods might either require significant time or leave students simply plugging numbers into equations that they don't understand. The advantages of using these simplifications must be weighed when deciding whether to use a more biologically accurate method. Again, a possible compromise might be to have students do the lesson as it is, but then explain to them the flaws in this methodology and have them compare their trees to ones built using more sophisticated algorithms [8]–[13].

### Future Directions

As mentioned above, the curriculum that we have developed is a first attempt to bring a computational element into the high school biology curriculum, not a final product. In future years of the eCSite project, the curriculum will be modified to take into account teaching experience as well as feedback from students, teachers, and members of the computational biology community. In addition, we encourage those who use the curriculum to modify and improve it (please tell us if you do, so others can benefit from your changes).

Our unit focuses on one of many areas of biology in which computers now play a significant role. Future lessons on computer modeling in areas such as epidemiology and ecology could be adapted from other eCSite units developed for middle school and high school classes [14]. New units could be developed on biological networks (metabolic networks, predator–prey relationships) and protein structure and function. On his blog, Professor Kevin Karplus of UC Santa Cruz has gone through the curriculum framework of the AP Biology course [15] and suggests several essential knowledge requirements that can be addressed in a computational manner. Dr. Karplus suggests that several requirements could be addressed using computational methods (this discussion can be found at http://gasstationwithoutpumps.wordpress.com/2011/01/08/advanced-placement-bio-changes-announced/). While our initial curriculum addressed some of these requirements, his analysis will prove valuable for planning new units.

In modifying lessons and developing new ones, we will have to keep in mind our goals. We want to introduce students to the connection between biology and computer science. We would like to show students computational techniques used in “real” biology while at the same time respecting the limits of their abilities: the students to whom this curriculum is directed have only an advanced high school knowledge of biology and little, if any, knowledge of computer science. Finally, we need to respect the limited amount of time available to devote to new content in a high school AP or IB Biology class.

A good unit for high school age students should be geared towards understanding existing algorithms rather than trying to build complex algorithms. Lessons should be based around understanding the algorithms used to solve biological problems and using that understanding to make better use of the algorithms.

## Conclusions

We incorporated a computational biology unit into advanced high school biology classes to teach students about the importance of computer science to biology. We presented a general introduction to algorithms, an overview of algorithms for searching for related DNA sequences (including BLAST), and algorithms for building phylogenetic trees.

Our unit enjoyed a great deal of success. Students got an idea of the volume of data involved in modern biological research and an appreciation of the need for computational methods to handle this data. They gained experience with a computational tool used by working biologists. Students also developed an appreciation for why biologists should have some understanding of how these computational tools work, even if they are not interested in developing new computational tools. High school teachers were able to independently incorporate the lessons they helped develop.

We encountered challenges associated with teaching high school students due to their lack of experience and the pressure they are under to pass high-stakes exams. A lesson geared towards students at this level needs to be compatible with their limited experience with mathematics and computer science and also be geared towards enhancing understanding of required material rather than replacing it. It should be made clear to students that they are learning the required curriculum using computational methods.

The lessons must be closely tied both to the existing local curriculum at the school and the broader AP or IB curriculum for advanced students. There should be connections between the computational lessons and past lessons in the class, as well as connections between the lessons and the high-stakes exams. To do this well, the regular teacher must be heavily involved.

The curriculum presented here is a work in progress. All activities are being evaluated to see how they further our goal of demonstrating the connection between computer science and biology. In future classes, some activities may be eliminated or modified to enhance our goals or to better reflect current biological research. This curriculum serves as a seed for further development, and our experiences teaching it can guide that development.

In future years of this project, we hope to improve the curriculum in response to student and teacher feedback (locally and from readers of this journal), as well as expanding it to include other areas of computational biology.
